# Genome-wide analysis of pain-, nerve- and neurotrophin -related gene expression in the degenerating human annulus

**DOI:** 10.1186/1744-8069-8-63

**Published:** 2012-09-10

**Authors:** Helen E Gruber, Gretchen L Hoelscher, Jane A Ingram, Edward N Hanley

**Affiliations:** 1 Department of Orthopaedic Surgery, Carolinas Medical Center, PO Box 32861, Charlotte, NC, 28232, USA; 2 Orthopaedic Research Biology, Cannon Research., Room 304, Carolinas Medical Center, PO Box 32861, Charlotte, NC, 28232, USA

**Keywords:** Low back pain, Neurotrophins, Nerves, Microarray analysis

## Abstract

**Background:**

In spite of its high clinical relevance, the relationship between disc degeneration and low back pain is still not well understood. Recent studies have shown that genome-wide gene expression studies utilizing ontology searches provide an efficient and valuable methodology for identification of clinically relevant genes. Here we use this approach in analysis of pain-, nerve-, and neurotrophin-related gene expression patterns in specimens of human disc tissue. Control, non-herniated clinical, and herniated clinical specimens of human annulus tissue were studied following Institutional Review Board approval.

**Results:**

Analyses were performed on more generated (Thompson grade IV and V) discs vs. less degenerated discs (grades I-III), on surgically operated discs vs. control discs, and on herniated vs. control discs. Analyses of more degenerated vs. less degenerated discs identified significant upregulation of well-recognized pain-related genes (bradykinin receptor B1, calcitonin gene-related peptide and catechol-*0*-methyltransferase). Nerve growth factor was significantly upregulated in surgical vs. control and in herniated vs. control discs. All three analyses also found significant changes in numerous proinflammatory cytokine- and chemokine-related genes. Nerve, neurotrophin and pain-ontology searches identified many matrix, signaling and functional genes which have known importance in the disc. Immunohistochemistry was utilized to confirm the presence of calcitonin gene-related peptide, catechol-0-methyltransferase and bradykinin receptor B1 at the protein level in the human annulus.

**Conclusions:**

Findings point to the utility of microarray analyses in identification of pain-, neurotrophin and nerve-related genes in the disc, and point to the importance of future work exploring functional interactions between nerve and disc cells in vitro and in vivo. Nerve, pain and neurotrophin ontology searches identified numerous changes in proinflammatory cytokines and chemokines which also have significant relevance to disc biology. Since the degenerating human disc is primarily an avascular tissue site into which disc cells have contributed high levels of proinflammatory cytokines, these substances are not cleared from the tissue and remain there over time. We hypothesize that as nerves grow into the human annulus, they encounter a proinflammatory cytokine-rich milieu which may sensitize nociceptors and exacerbate pain production.

## Background

Low back pain brings the patient to the spine surgeon, but the relationship between disc degeneration and pain production in the disc is still poorly understood. Patients with chronic low back pain do not have the leg pain which results when fragments of herniated disc push on nerves; instead, these patients experience pain that is thought to arise from the disc itself. Although some back pain may be related to lumbosacral anatomy/function spinal anatomy [1], age, and other factors (see Fairbank et al. for a recent systematic review of low back pain classification [2]), discogenic low back pain is not well understood [3-8]. As with all pain, the pain-initiating event results from complex cellular, molecular and functional events at the nociceptors (naked nerve endings) [9,10]. Discogenic pain is believed to result from disc changes (possibly from outer annulus pressure on nerve endings, disc cell dysfunction, products of matrix degradation [11], or unknown events) which influence the nervous system by stimulation of annulus nociceptors.

Recent studies focused over the last decade on molecular events and gene expression patterns related to pain [12], and studies have revealed that genome-wide gene expression studies provide a powerful methodology for identification of clinically relevant genes [13]. The objective of the present study was to perform a genome-wide analysis of annulus tissue from patients with discogenic back pain, compared to disc tissue from control subjects and herniated disc patients, in an analysis of the expression of pain, nerve and neurotrophin-related genes. Ontology searches for these specific topics were utilized in order to avoid searching large gene array data bases gene by gene, and because this technique provides a controlled vocabulary of search terms for gene characteristics [14].

As cell-based therapies for disc degeneration progress, information on pain-, nerve- and neurotrophin-gene expression in disc tissue becomes increasingly important. Findings presented here have potential applications in future treatment modalities, such as perispinal administration of TNF-α inhibitors (such as etanercept/enbrel [15]) or use of specific small molecular antagonists to neurotrophins. New information presented here on the relationship between proinflammatory mediators, nerves and neurotrophins has the potential to contribute to future important antagonist profiles with application to discogenic back pain.

## Results

Demographic features of the patient population are summarized in Table 1 which presents spinal site, grade, subject age, and whether surgical specimens were derived from herniated or non-herniated tissue. In the present work, gene expression analyses included three Thompson grade II and five grade III control disc specimens (obtained from Cooperative Human Tissue Network). Surgical specimens were analyzed from three grade II discs (two specimens of which were from herniated discs), four grade III discs (from herniated discs), five grade IV specimens (two of which were from herniated discs), and three grade V specimens (two of which were from herniated discs).

**Table 1:** Demographic data on disc tissues*

**Subject number**	Site*	**Thompson grade**	**Age (years)/gender**	**Herniated?**	**Other information**
1	Lumbar	II	34/F	No	Control
2	L3-L4	II	30/M	No	Control
3	L4-L5	II	54/F	No	Surgical specimen
4	Lumbar	II	34/F	No	Control
5	L5-S1	II	21/M	Yes	Surgical specimen; immuno **
6	L4-L5	II	40/F	Yes	Surgical specimen
7	L4-L5	III	36/F	Yes	Surgical specimen
8	C6-C7	III	45/F	Yes	Surgical specimen
9	L3-L4	III	52/F	No	Control
10	L3-L4	III	52/F	No	Control; immuno
11	L3-L4	III	52/F	No	Control
12	L3-L4	III	52/F	No	Control
13	L2-L3	III	33/F	No	Control
14	L5-S1	III	37/M	Yes	Surgical specimen; immuno
15	L4-L5	III	43/M	Yes	Surgical specimen; immuno
16	L5-S1	IV	63 F	No	Surgical specimen
17	L2-L3	IV	65/F	No	Surgical specimen
18	C5-C6	IV	59/M	Yes	Surgical specimen
19	L5-S1	IV	45/M	Yes	Surgical specimen; immuno
20	L5-S1	IV	43/M	No	Surgical specimen
21	L5-S1	V	72/F	Yes	Surgical specimen; immuno
22	L4-5	V	41/M	No	Surgical specimen; immuno
23	C6-7	V	57/F	Yes	Surgical specimen

* Note that multiples specimens may have been derived from some subjects. L, lumbar; C, cervical; M, male; F, female; Control discs are normal discs obtained from the Cooperative Human Tissue Network.

** Indicates that specimens were utilized for selected immunohistochemical studies.

The ontology gene expression analysis for pain and nerve categories included a variety of biological processes, molecular functions, and cellular components. To assist the reader in understanding these, Table 2 outlines the categories included in our searches.

**Table 2:** Outline of ontology search strategies


**Pain Ontologies:**
A. Biological Process:
1. Sensory perception
a. Sensory perception of pain:
I. Regulation of sensory perception of pain
II. Detection of chemical stimulus involved
III. Detection of temperature stimulus involved
IV. Detection of mechanical stimulus involved
2. Ion transport
3. Inflammatory response
4. Behavior
5. Response to pain:
a. Behavioral response to pain
6. G-protein coupled receptor (protein) signaling pathway
7. Transmission of nerve impulse
8. Fatty acid catabolic process
B. Molecular Function:
1. 1. Carboxyl- or carbamoyl-transferase activity
2. Opioid peptide activity
3. Signal transducer activity
a. G-protein coupled receptor activity
b. Galanin receptor activity
c. Bradykinin receptor activity
d. Opioid receptor activity
e. Adrenergic receptor activity
4. Voltage-gated channel activity
5. G-protein coupled receptor binding
C. Cellular component
1. Plasma lipoprotein particle
2. Axon
**Nerve Ontologies:**
A. Biological Process:
1. Neuron death
a. Neuron apoptosis
i. Positive regulation of neuron apoptosis
ii. Negative regulation of neuron apoptosis
a. Neurotrophin production
iii. Neuroprotection
2. Neuropeptide signaling pathway	
3. Transmission of nerve impulse	
4. Nervous system development:	
a. Nerve development	
b. Neurogenesis	
i. Regulation of neurogenesis	
a. Positive regulation of neurogenesis	
b. Negative regulation of neurogenesis	
ii. Neuron differentiation	
a. Neuron projection development	
i. Axonogenesis regulation:	
a. Positive regulation of axonogenesis	
b. Negative regulation of axonogenesis	
iii. Neuroblast differentiation	
iv. Neuroblast proliferation	
v. Neuron migration	
B. Molecular function:	
1. Neurotrophin binding:	
a. Nerve growth factor binding	
2. Neurotrophin receptor activity	
3. Neurotransmitter receptor activity	
a. Substance P receptor activity	
4. Calcitonin receptor binding	
5. Vascular endothelial growth factor (-activated) receptor activity	
6. Protein kinase activity	
7. Neuropeptide hormone activity	
8. Neuropeptide binding	
9. Ciliary neurotrophic factor receptor binding	
10. Neurotrophin receptor binding:	
a. Nerve growth factor receptor binding	
C. Cellular component:	
1. Neuron projection:	
a. Axon	
b. Dendrite	
2. Endoplasmic reticulum lumen	
3. Nuclear outer membrane	

In each of our summary tables on gene expression findings (Tables 3, 4 and 5), genes are listed which have relevance to nerves, pain and neurotrophins (listed in section A of Tables 3, 4 and 5), genes with relevance to proinflammatory cytokines and chemokines (listed in section B of Tables 3, 4 and 5) and genes with specificity not only to nerve, pain and neurotrophins but also to the disc itself (listed in section C of the tables).

**Table 3:** Significant differences in pain-, neurotrophin-, nerve-, and disc-related genes in more degenerated discs (Grades iv and v) compared to less degenerated discs (Grades i – iii)

**Gene name**	**Gene identifier**	**Fold change**	**Direction**	**p-value**
***A. Pain-, Neurotrophin- and Nerve-related Genes***				
Adrenergic, beta, receptor kinase 2	AI478542	1.16	Up	0.045
Ataxin 10	AF119662	2.62	Up	0.008
Bradykinin receptor B1	NM_000710	1.09	Up	0.023
Calcitonin gene related peptide	AI478743	1.51	Up	0.035
Catechol-O-methyltransferase	NM_001670	1.17	Up	0.020
Cholinergic receptor, muscarinic 1	AI500293	1.60	Down	0.042
Clusterin	M25915	12.76	Up	0.010
EGF, latrophilin and seven transmembrane domain containing 1	NM_022159	1.74	Up	0.040
Endoplasmic reticulum aminopeptidase 2	BE889628	1.28	Up	0.036
Endoplasmic reticulum protein 44	BC005374	2.08	Up	0.043
GABA binding protein 2	BC002557	4.54	Down	0.027
GABA(A) receptor-associated protein	NM_007278	3.78	Up	0.049
Gap junction protein, alpha 1, 43 kDa	NM_000165	4.91	Up	0.017
Glutathione peroxidase 1	NM_000581	3.42	Up	0.036
Glutamate receptor, metabotropic 1	U31216	1.06	Up	0.0082
Guanine nucleotide binding protein (G protein), gamma 10	NM_004125	2.97	Up	0.049
Guanine nucleotide binding protein (G protein) alpha inhibiting activity polypeptide 3	J03198	3.22	Up	0.021
Guanine nucleotide binding protein (G protein) beta polypeptide 1	NM_002074	4.04	Up	0.015
Heat shock protein 90 kDa alpha (cytosolic), blass B member 1	AF275719	2.96	Up	0.038
Hydroxysteroid (17-beta) dehydrogenase 4	NM_000414	1.88	Up	0.049
Kv channel interacting protein 2	AF367019	1.52	Up	0.027
Neurogenin 2	AF022859	1.08	up	0.045
Neuron navigator 1	N57538	2.42	Up	0.009
Neuron navigator 2	AA011020	1.11	Up	0.043
Neuropilin 2	AA295257	1.87	Up	0.037
Neuroplastin	NM_017455	2.78	Up	0.048
Opioid growth factor receptor	AF172449	1.31	Up	0.008
Palmitoyl-protein thioesterase 1	NM_000310	3.04	Up	0.019
PDZ and LIM domain 5	AV715767	5.1	Up	0.006
Peroxisome proliferator-activated receptor delta	NM_006238	3.31	Down	0.031
Phytanoyl-CoA 2-hydroxylase	NM_006214	2.17	Up	0.049
Potassium channel tetramerisation domain containing 10	AS073741	2.69	Up	0.036
Potassium channel tetramerisation domain containing 15	W73820	1.33	Up	0.013
Potassium voltage-gated channel, delayed-rectifier, subfamily S, member 3	NM_002252	1.66	Up	0.031
Presenilin 1	NM_007318	1.61	Up	0.024
Regulator of G-protein signaling 5	AF159570	2.31	Up	0.047
Reticulon 4 (Neurite growth inhibitor)	AF20999	4.61	Up	0.001
Solute carrier family 38, member 1	NM_030674	2.13	Up	0.039
Solute carrier family 7 (catonic amino acid transporter, y + system), member 1	AA148507	1.44	Up	0.017
Somatostatin receptor 4	NM_001052	1.09	Down	0.033
S100 calcium binding protein A4	NM_002961	7.44	Up	0.010
Thioredoxin domain containing 12 (endoplasmic reticulum)	AF131758	2/42	Up	0.009
Thy-1 cell surface antigen	AL161958	3.48	Up	0.006
***B. Proinflammatory cytokine and chemokine genes:***				
Chemokine (C-X-C motif) receptor 7	AI817041	3.32	Up	0.043
Chemokine (C-X-C motif) ligand 12 (stromal cell-derived factor 1)	NM_000609	1.90	Up	0.024
FGF receptor 2	T83672	1,12	Up	0.020
IL-17A	NM_002190	1.05	Up	0.048
Interferon induced transmembrane protein 1 (9–27)	NM_003641	2.72	Up	0.026
PDGF C	NM_016205	3.07	Up	0.041
PDGF alpha polypeptide	NM_006206	5.73	Up	0.021
PDGF receptor, beta polypeptide	NM_003609	2.10	Up	0.033
PDGF receptor-like	NM_006207	1.82	Up	0.037
TGF beta	NM_000358	6.68	Up	0.035
Latent TGF beta binding protein 1	NM_000627	3.29	Up	0.016
TNF receptor-associated factor 5	NM_004619	1.34	Up	0.014
VEGF B	NM_003377	1.09	Up	0.037
***C. Genes with special disc relevance:***				
ADAM metallopeptidase domain 17	NM_003183	1.56	Up	0.001
Apoptosis-inducing factor, mitochondrion associated, 1	NM_004208	1.42	Up	0.019
BMP receptor, type II (serine/threonine kinase)	AI457436	1.51	Up	0.027
Calcitonin receptor-like	AI1478743	1.51	Up	0.035
Caspase 2, apoptosis-related cysteine peptidase	AU153405	1.63	Up	0.028
Caspase 6, apoptosis-related cysteine peptidase	BC000305	1.63	Up	0.048
Collagen, type I, alpha 1	K01228	2.69	Up	0.046
Collagen, type I, alpha 2	NM_000089	13.27	Up	0.007
Collagen type III, alpha 1	AU144167	10.3	Up	0.036
Collagen type IV, alpha 2	X05610	2.02	Up	0.021
Collagen type V, alpha 1	AI9833428	1.5	Up	0.039
Collagen type VI, alpha 3	NM_004369	5.85	Up	0.036
Connective tissue growth factor	M92934	6.47	Up	0.009
EGF-containing fibulin-like extracellular matrix protein 1	NM_004105	1.49	Up	0.005
Fibronectin 1	AK026737	16.82	Up	0.017
Fibronectin leucine rich transmembrane protein 2	NM_013231	2.78	Up	0.023
Hypoxia inducible factor 1, alpha subunit	NM_001530	3.61	Up	0.037
Janus kinase 2	AF001362	1.3	Up	0.002
Laminen, alpha 5	BC003355	1.39	Up	0.042
Laminin, beta 1	NM_002291	2.13	Up	0.045
Lumican	NM_002345	17.58	Up	0.015
Mitogen-activated protein kinase 1	AF320999	4.61	Up	0.0017
Mitogen-activated protein kinase 13	BC000433	1.37	Up	0.014
Mitogen-activated protein kinase kinase kinase 3	BF971923	1.67	Up	0.045
Nitric oxide synthase 3	NM_000603	4.77	Down	0.030
SPARC (osteonectin), cwcv and Kazal-like domains proteoglycan (testican) 1	AF231124	2.74	Up	0.027
Thrombospondin 1	BF055462	1.20	Up	0.033
TIMP metallopeptidase inhibitor 2	BF107565	3.57	Up	0.034
TIMP metallopeptidase inhibitor 3	U67195	2.88	Up	0.035
Vimentin	AI520969	1.35	Up	0.031

**Table 4:** Significant differences in pain-, neurotrophin-, nerve- and disc related gene expression in surgical compared to control (CHTN) specimens

**Gene name**	**Gene identifier**	**Fold change**	**Direction**	**p-value**
***A. Pain-, Neurotrophin-, and Nerve-related genes:***				
Adrenergic, beta, receptor kinase 1	NM_001619	1.28	Down	0.015
Ataxin 1	NM_000332	1.89	Up	0.026
Ataxin 10	AF119662	2.43	Up	0.016
Caireticulin	NM_004343	8.53	Up	0.005
Calcitonin gene-related peptide	M26095	1.21	Down	0.005
Calcium channel, voltage-dependent, gamma subunit 2 (stargazin)	NM_006078	1.18	Down	0.037
Calcium channel, voltage-dependent, P/Q type, alpha 1A subunit	AA769818	1.92	Up	0.023
Calcium channel, voltage-dependent, beta 4 subunit	NM_000726	1.24	Down	0.001
Calmodulin 3 (phosphorylase kinase, delta)	NM_005184	4.29	Up	0.002
Calreticulin	NM_004343	8.53	Up	0.005
Cannabinoid receptor 1 (brain)	NM_001840	1.14	Down	0.021
Catechol-*O*-methyltransferase	AW139431	1.29	Down	0.035
Chloride intracellular channel 1	L08666	3.61	Up	0.035
Cholinergic receptor, muscarinic 1	AI500293	1.99	Down	0.001
Ciliary neurotrophic factor	NM_000614	1.14	Down	0.040
Clusterin	AI982754	20.62	Up	0.008
Early growth response 1	NM_001964	5.73	Up	0.013
Family with sequence similarity 123, member B	NM_019000	3.45	Up	0.022
Frizzled homolog 8	AB043703	2.81	Up	0.016
G protein-coupled receptor, family C, group 5, member C	NM_022036	2.32	Up	0.039
G protein-coupled receptor 87	NM_023915	1.17	Down	0.006
G protein-coupled receptor 98	AF037334	1.17	Down	0.043
G protein regulated inducer of neurite outgrowth 1	AI052709	1.05	Down	0.046
GABA binding protein 2	BC002557	8.78	Down	0.0006
GABA(A) receptor-associated protein	NM_007278	4.04	Up	0.038
GABA A receptor, beta 2	NM_021911	1.2	Down	0.006
Gap junction protein, alpha 1, 43 kDa	NM_000165	4.1	Up	0.038
Glutamate receptor, ionotropic, delta 2	NM_001510	1.17	Down	0.046
Glutamate receptor, ionotropic, kainite 5	S40369	1.28	Down	0.009
Glutamate receptor, ionotropic, N-methyl D-aspartate 2B	NM_024016	1.08	Down	0.017
Glutamate receptor, metabotropic 5	D60132	1.38	Down	0.007
Guanine nucleotide binding protein (G protein), alpha inhibiting activity polypeptide 3	J03198	2.87	Up	0.040
Guanine nucleotide binding protein (G protein), beta polypeptide 1	NM_002074	4.36	Up	0.009
Guanine nucleotide binding protein (G protein), alpha 11 (Gq class)	NM_002067	1.29	Up	0.024
Glutathione peroxidase 1	NM_000581	3.62	Up	0.028
Low density lipoprotein-related protein 1	NM_002332	5.26	Up	0.034
Meteorin, glial cell differentiation regulator	BE965311	1.28	Down	0.049
Natriuretic peptide receptor B/guanylate cyclase B	NM_003995	1.55	Up	J0.033
Nerve growth factor, beta polypeptide	NM_002506	1.44	Up	0.03
Netrin 1	BF591483	1.16	Down	0.041
Neugrin, neurite outgrowth associated	AL037339	2.66	Up	0.028
Neuroligin 1	AI912122	1.13	Down	0.043
Neuron navigator 1	AB032977	3.83	Up	0.005
Neuro-oncological ventral antigen 1	NM_002515	3.1	Up	0.0008
Neuropilin 2	BC009222	3.28	Up	0.014
Neuroplastin	NM_017455	3.61	Up	0.015
Neurotensin	NM_006183	1.22	Down	0.009
Nexin (serpin peptidase inhibitor, claude E)	AL541302	5.64	Up	0.009
Oligophrenin 1	NM_002547	2.82	Up	0.048
Opioid growth factor receptor-like 1	BE500942	2.4	Up	0.023
Palmitoyl-protein thioesterase 1	NM_000310	3.64	Up	0.005
PDZ and LIM domain 5	AV15767	5.06	Up	0.006
Potassium large conductance calcium-activated channel, subfamily M, alpha member 1	U11058	3.44	Up	0.040
Potassium voltage-gated channel, shaker-related subfamily, member 10	NM_005549	2.02	Down	0.010
Potassium channel tetramerisation domain containing 20	AV707142	2.34	Up	0.018
Prepronociceptin	NM_006228	1.04	Down	0.027
Regulator of G-protein signaling 3	NM_021106	2.45	Up	0.039
Regulator of G-protein signaling 12	AF030111	1.18	Down	0.006
Regulator of G-protein signaling 18	AF076642	1.3	Down	0.025
Reticulon 4 (Neurite growth inhibitor)	AB015639	5.96	Up	0.004
Roundabout, axon guidance receptor, homolog 3	NM_022370	1.89	Up	0.025
S100 calcium binding protein A4	NM_002961	12.85	Up	0.003
S100 calcium binding protein A6	NM_014624	6.97	Up	0.013
S100 calcium binding protein B	BC001766	3.48	Up	0.009
Sigma non-opioid intracellular receptor 1	NM_005866	1.64	Up	0.031
Solute carrier family 1 (glutamate/neutral amino acid transporter), member 4	W72527	4.06	Up	0.001
Solute carrier family 29 (nucleoside transporters), member 1	NM_004955	2.89	0.009	
Solute carrier family 38, member 1	NM_030674	2.39	Up	0.015
Synaptopodin	NM_007286	2.24	Up	0.032
Thioredoxin domain containing 5 (endoplasmic reticulum)	NM_030810	6.05	Up	0.002
Thy-1 cell surface antigen	AL161958	3.13	Up	0.013
Voltage-dependent anion channel 2	L08666	3.61	Up	0.035
Xylosyltransferase 1	AI693140	6.23	Up	0.004
***B. Proinflammatory cytokine and chemokine genes:***				
Chemokine (C-X-C motif) ligand 2	AV648479	1.15	Down	0.027
Chemokine (C-X-C motif) receptor 7	AI817041	4.13	Up	0.014
FGF 1 (acidic)	X59065	2.17	Up	0.033
FGF 5	AB016517	1.47	Down	0.015
FGF receptor substrate 2	AI708648	1.23	Down	0.027
FGF receptor 3	NM_000142	2.62	Up	0.048
FGF receptor substrate 2	AI708648	1.23	Down	0.027
IL-1 receptor-associated kinase 1	NM_001569	3.98	Up	0.010
IL-1 receptor-like 1	NM_003856	1.16	Down	0.041
IL-31 receptor B1	NM_139017	1.17	Down	0.025
IL-6 signal transducer (gp130, oncostatin M receptor)	AW242916	1.56	Down	0.032
Interferon gamma receptor 2	NM_005534	2.25	Up	0.041
Macrophate migration inhibitory factor	NM_002415	5.71	Up	0.004
PDGF C	NM_016205	4.62	Up	0.003
PDGF receptor, alpha polypeptide	NM_006206	6.63	Up	0.011
TGF beta	NM_000358	8.77	Up	0.014
TGF beta receptor 1	AA604375	3.14	Up	0.023
TNF (ligand) superfamily, member 11	AF053712	3.28	Up	0.009
TNF alpha-indued protein 6	AW188198	9.91	Up	0.006
TNF receptor superfamily member 1A	NM_001065	2.01	Up	0.040
TNF receptor superfamily, member 10c, decoy without an intracellular domain	AK026079	1.34	Down	0.014
TNF receptor superfamily, member 10d, decoy without an intracellular domain	NM_003841	1.18	Down	0.039
TNF receptor superfamily, member 12A	NM_016639	2.01	Up	0.046
TNF receptor superfamily, member 25	U94506	1.16	Down	0.012
TNF receptor-associatee factor 3	AI721219	1.57	Up	0.046
***C. Genes with special disc relevance:***				
ADAM metallopeptidase domain 17	NM_003183	1.38	Up	0.025
ADAM metallopeptidase domain 22	NM_021723	1.18	Down	0.045
BCL2-associated athanogene 5	NM_004873	1.9	Up	0.028
BMP and activin membrane-bound inhibitor homolog	NM_012342	3.28	Up	0.008
Brevican	AA622130	1.30	Down	0.044
Caspase 6, apoptosis-related cysteine peptidase	BC000305	2.05	Up	<0.0001
Collagen type II, alpha 1	X)6268	53.83	Up	0.009
EGF-containing fibulin-like extracellular matrix protein 2	NM_005507	11.88	Up	0.012
FGF 5	AB016517	1.47	Down	0.015
Fibronectin 1	AK026737	37.09	Up	0.001
Growth arrest specific 1	NM_002048	5.44	Up	0.006
Growth arrest specific 7	BE439987	2.56	Up	0.040
Heat shock 70kDa protein 5	AW052044	1.34	Up	0.047
Heat shock 70kDa protein 8	AA704004	3.99	Up	0.023
Heat shock protein 90 kDa beta, (group 94), member 1	AK025862	2.45	Up	0.031
Hypoxia inducible factor 1, alpha subunit	NM_001530	4.41	Up	0.014
Laminin, beta 2	X79683	3.24	Up	0.029
Latent TGF beta binding protein 1	NM_000627	3.09	Up	0.024
Lumican	NM_002345	66.35	Up	0.0001
Lysyl oxidase-like 2	NM_002318	5.06	Up	0.010
Mitogen-activated protein kinase 1	BF434653	1.38	Down	0.018
Mitogen-activated protein kinase 3	X60188	1.94	Up	0.030
Mitogen-activated protein kinase kinase kinase 3	BF971923	1.83	Up	0.016
Mitogen-activated protein kinase 13	BC000433	1.35	Up	0.020
Mitogen-activated protein kinase 14	AA604375	3.14	Up	0.023
Proteoglycan 4	NM_005807	13.72	Up	0.001
PTH 1 receptor	NM_000316	1.77	Up	0.047
Retinoic acid receptor, beta	NM_000965	1.61	Up	0.036
SOD	NM_000454	4.18	Up	0.023
SPARC/osteonectin, cwcv and kazal-like domains proteoglycan (testican) 1	AF231124	3.00	Up	0.015
Tenascin R	Y13359	1.38	Down	0.014
Thyroid stimulating hormone receptor	BE045816	1.26	Down	0.019
TIMP metallopeptidase inhibitor 3	NM_000362	12.35	Up	0.001
TIMP metallopeptidase inhibitor 4	NM_003256	2.43	Up	0.042
Versican	BF218922	5.19	Up	0.021

**Table 5:** Significant differences in pain-, neurotrophin-, nerve-related and disc genes in herniated discs compared to control (CHTN) discs

**Gene name**	**Gene identifier**	**Fold change**	**Direction**	**p-value**
***A. Pain-, Neurotrophin-, Nerve-related Genes:***				
Adrenergic, beta, receptor kinase 1	NM-001619	1.33	Down	0.042
Ataxin 1	NM_000332	2.09	Up	0.031
Calcitonin gene related peptide	M26095	1.2	Down	0.040
Calcium channel, voltage-dependent, P/Q type, alpha 1A subunit	AA769818	2.17	Up	0.018
Calcium channel, voltage-dependent, beta 2 subunit	U80764	1.21	Up	0.037
Cannabinoid receptor 1 (brain)	NM-001840	1.16	Down	0.029
Chemokine (C-X-C motif) receptor 7	AI817041	4.51	Up	0.012
Cholinergic receptor, muscarinic 1	AI500293	2.05	Down	0.003
Clusterin	M25915	20.02	Up	0.003
Corticotropin releasing hormone receptor 1	X72304	1.53	Up	0.035
EPH receptor B3	NM_004443	1.52	Up	0.037
Family with sequence similarity 134, member B	NM_019000	3.72	Up	0.021
GABA(A) receptor-associated protein	NM_007278	4.39	Up	0.039
GABA A receptor, beta 2	NM_021911	1.21	Down	0.016
G protein-coupled receptor, family C, group 5, member C	NM_022036	2.64	Up	0.036
G protein-coupled receptor 52	NM_005684	1.06	Down	0.044
G protein-coupled receptor 161	AI703188	1.71	Up	0.044
G protein signaling modulator 2 (AGS3-like)	AW195581	1.94	Up	0.040
Glutamate receptor, metabotropic 5	D60132	1.37	Down	0.026
Glutaminase	NM_014905	3.14	Up	0.014
Glutathione peroxidase 1	NM_000581	3.94	Up	0.038
Guanine nucleotide binding protein (G protein), beta polypeptide 1	NM_002074	4.61	Up	0.013
Guanine nucleotide binding protein (G protein), gamma 5	NM_005274	2.62	Up	0.040
Guanine nucleotide binding protein (G protein), gamma 7	AL039870	1.54	Up	0.006
Guanine nucleotide binding protein (G protein), gamma 10	AI765321	1.49	Up	0.040
Guanine nucleotide binding protein (G protein), gamma 11	NM_004126	3.13	Up	0.026
Guanine nucleotide binding protein (G protein), gamma 12	N32508	1.86	Up	0.026
Guanine nucleotide binding protein (G protein), alpha 13	AI928136	2.80	Up	0.022
Kallidrein-related peptidase 8	NM_144506	1.21	Down	0.028
Monoglyceride lipase	BG168471	1.79	Up	0.019
Myelin basic protein	N37023	3.28	Up	0.003
Natriuretic peptide receptor B/guanylate cyclase B (atrionatriuretic peptide receptor B)	NM_003995	2.14	Up	0.049
Neogenin homolog 1	BF058828	1.28	Down	0.022
Nerve growth factor (beta polypeptide)	NM_002506	1.5	Up	0.029
Neural precursor cell expressed, developmentally downregulated 4-like	AB007899	1.23	Up	0.038
Neuromedin U receptor 2	AF272363	1.09	Down	0.023
Neuro-oncological ventral antigen 1	NM_002515	3.1	Up	0.001
Neurofibromin 1	M60915	1.04	Up	0.031
Neuromedin U receptor 2	AF272363	1.09	Down	0.023
Neuropilin 2	AA295257	3.92	Up	0.032
Neuroplastin	NM_017455	4.46	Up	0.008
Opioid growth factor receptor-like 1	BE500942	2.52	Up	0.017
Palmitoyl-protein thioesterase 1	NM_000310	4.34	Up	0.005
Pancreatic polypeptide receptor 1	U42387	1.31	Down	0.048
PDZ and LIM domain 5	AV715767	5.04	Up	0.007
Plexin A1	T16388	1.51	Down	0.046
Potassium voltage-gated channel, shaker-related subfamily, member 10	NM_005549	2.36	Down	0.007
Prostaglandin E receptor 2 (subtype EP2), 53 kDa	NM_000956	1.45	Up	0.048
Regulator of G-protein signaling 3	NM_021106	3.15	Up	0.021
Regulator of G-protein signaling 12	AF030111	1.18	Down	0.027
Regulator of G-protein signaling 14	NM_006480	1.13	Up	0.042
Reticulon 4 (neurite growth inhibitor)	AB015639	5.58	Up	0.007
Roundabout, axon guidance receptor, homolog 3	NM_022370	1.74	Up	0.048
S100 calcium binding protein A6	NM_014624	9.13	Up	0.017
S100 calcium binding protein A9	NM_002965	1.93	Up	0.046
S100 calcium binding protein B	BC001766	4.18	Up	0.012
Sigma non-opioid intracellular receptor	NM_005866	2.01	Up	0.022
Solute carrier family 1 (glutamate/neutral amino acid transporter), member 4	AI889380	2.79	Up	0.013
Solute carrier family 6 (neurotransmitter transporter, creatine), member 8	AI820043	1.65	Down	0.021
Solute carrier family 22, member 17	NM_020372	2.62	Up	0.044
Solute carrier family 29 (nucleoside transporters), member 1	NM_004955	4.03	Up	0.010
Solute carrier family 38, member 1	NM_030674	2.25	Up	0.023
Solute carrier family 38, member 2	NM_018976	2.24	Up	0.048
Solute carrier family 44, member 2	AI264216	1.88	Up	0.020
Syntaxin 1A (brain)	NM_004603	2.46	Up	0.017
Syntaxin binding protein 1	NM_003165	1.96	Up	0.049
Thy-1 cell surface antigen	NM_000633	2.24	Up	0.025
Xylosyltransferase I	AI693140	6.41	Up	0.007
***B. Proinflammatory cytokine and chemokine genes:***				
Chemokine (C-C motif) ligand 22	NM_002990	1.10	Down	0.039
IL 11 receptor, alpha	NM_147162	1.03	Down	0.049
IL 17 receptor C	BF196320	1.11	Down	0.033
IL 27 receptor, alpha	AI983115	1.19	Down	0.042
IL 31 receptor A	AF106913	1.33	Down	0.040
IL-1 receptor-associated kinase 1	NM_001569	4.30	Up	0.015
IL-23, alpha subunit P19	AJ296370	1.62	Up	0.023
Interferon gamma receptor 2	NM_005534	2.79	Up	0.028
Latent TGF beta binding protein 1	NM_000627	2.61	Up	0.039
Macrophage migration inhibitory factor	NM_002415	5.01	Up	0.034
PDGF receptor, alpha polypeptide	NM_006206	5.96	Up	0.018
TGF beta	NM_000358	12.34	Up	0.011
TGF beta receptor 1	NM_000714	3.88	Up	0.018
TNF alpha-induced protein 6	AW188198	14.57	Up	0.002
TNF receptor superfamily, member 11b	NM_002546	3.31	Up	0.034
TNF receptor superfamily, member 12A	NM_016639	2.29	Up	0.042
TNF receptor superfamily, member 25	U94506	1.13	Down	0.046
TNF receptor-associated factor 3	AI721219	1.67	Up	0.043
TNF superfamily, member 11	AF053712	4.32	Up	0.002
***C. Genes with special relevance to the disc:***				
ADAM metallopeptidase domain 17	NM_003183	1.36	Up	0.045
BCL2-associated athanogene 5	NM_004873	1.95	Up	0.039
Caspase 6, apoptosis-related cysteine peptidase	BC000305	2.48	Up	0.025
Collagen type I, alpha 2	NM_000089	11.05	Up	0.017
Collagen type II, alpha 1	X06268	58.92	Up	0.0001
Collagen type III, alpha 1	AU144167	12.15	Up	0.026
Collagen type VI, alpha 2	AL531750	3.14	Up	0.018
Collagen type VI, alpha 3	NM_004369	8.55	Up	0.013
Collagen type VI, alpha 1	AA292373	3.12	Up	0.037
Collagen type XI, alpha 1	NM_001854	6.45	Up	0.020
Collagen type IX, alpha 3	NM_001853	10.38	Up	0.002
EGF receptor pathway substrate 8	NM_004447	1.55	Up	0.048
EGF-containing fibulin-like extracellular matrix protein 2	NM_005507	9.96	Up	0.037
Fibronectin 1	AK026737	52.43	Up	0.0004
Fibronectin leucine rich transmembrane protein 2	NM_013231	2.61	Up	0.028
Growth arrest-specific 1	NM_002048	5.45	Up	0.006
Growth arrest-specific 7	NM_005890	2.04	Up	0.045
Heat shock 70 kDa protein 8	AA704004	5.36	Up	0.008
Hypoxia indicuble factor 3, alpha subunit	NM_001530	4.32	Up	0.027
Hypoxia inducible factor 1 (HIF1A)	NM_001530	4.32	Up	0.027
Jun oncogene	NM_002228	2.21	Up	0.027
Lumican	NM_002345	79.38	Up	<0.0001
Lysly oxidase-like 2	NM_002318	5.85	Up	0.009
Mitogen activated protein kinase 1	BF434653	1.44	Down	0.022
Mitogen-activated protein kinase 3	X60188	2.28	Up	0.023
Mitogen-activated protein kinase 8	AU152505	1.41	Up	0.048
Mitogen-activated protein kinase 13	BC000433	1.32	Up	0.030
Mitogen-activated protein kinase 14	NM_001315	4.30	Up	0.006
Never in mitosis gene a (NIMA)-related kinase 8	AI9365173	1.81	Up	0.006
Never in mitosis gene a (NIMA)-related kinase 7	AL080111	2.43	Up	0.039
Proteoglycan 4	NM_005807	18.05	Up	0.001
SOD 1, soluble	NM_000454	4.44	Up	0.038
SPARC/osteonectin, cwcv and kazal-like domains proteoclycan (testican) 1 (SPOCK1)	AF231124	3.3	Up	0.010
Thyrotropin-releasing hormone receptor	D16845	1.25	Down	0.004
TIMP metallopeptidase inhibitor 2	BF107565	2.9	Up	0.046
TIMP metallopeptidase inhibitor 3	NM_000358	15.36	Up	0.002
Versican	BF218922	6.59	Up	0.013
Vitamin D receptor	AA454701	1.19	Down	0.039

### Neurotrophin-, nerve-, and pain-related gene, and disc gene expression patterns in the annulus: expression patterns in more degenerate compared to less degenerate discs

In Table 3, findings are presented for selected relevant genes which were significantly elevated in expression in more degenerated Thompson grade IV and V discs compared to findings in grades I, II and III discs.

Of major interest in Table 3 are a number of genes with high relevance to pain, neurotrophins and nerves. These include significantly upregulated expression in the more degenerated discs of the following: bradykinin receptor B1, calcitonin gene-related peptide, catechol-O-methyltransferase, neuron navigator-1 and −2, neuropilin 2, and reticulon 4 (also known as neurite growth inhibitor). These genes showed up regulation fold changes ranging from 1.09 to 4.61.

A large number of genes in the ion transport grouping showed significant changes in this analysis; 69 genes showed significant up regulation, and 59 significant down regulation (most data not shown).

Genes with specific high relationships to disc cell biology included these significantly upregulated genes in the more degenerated discs: apoptosis-inducing factor (mitochondrion associated), BMP receptor, type II, collagens type I, III, IV, V and VI, connective tissue growth factor, fibronectin, hypoxia inducible factor 1 (HIF1), several of the mitogen-activated protein kinases, SPARC (osteonectin), TGF-ß, lumican and TIMP metallopeptidase inhibitors-2 and −3. These genes showed upregulation fold changes ranging from 1.42 to 17.58. Nitric oxide synthase 3 showed a 4.77 fold downregulation.

### Neurotrophin-, nerve-, and pain-related gene, and disc gene expression patterns in the annulus: analysis of expression patterns in surgical compared to control (CHTN) specimens

In Table 4, findings are presented for relevant genes which were significantly upregulated in surgically operated disc specimens compared to expression findings in control (CHTN) discs.

Of high interest in Table 4 are a number of genes with high relevance to pain, neurotrophins and nerves. These include the following: calcitonin gene-related peptide, catechol-O-methyltransferase, ciliary neurotrophic factor, nerve growth factor, neuron navigator 1, neuropilin 2, reticulon 4, and roundabout axon guidance receptor. These genes showed up regulation fold changes ranging from 1.2 to 5.96.

A large number of genes in the ion transport grouping also showed significant changes in this analysis; 87 genes showed significant up regulation, and 73 significant down regulation (most of the gene data in the ion transport group are not shown here).

Genes with specific high relationships to disc cell biology included these significantly upregulated genes in surgical vs. control discs: heat shock proteins, fibronectin 1, versican, lumican, several of the mitogen-activated protein kinases, TIMP metallopeptidase inhibitors-3 and −4, TFG-ß, latent TGF binding protein 1, several of the TNF receptors, and collagen type II alpha 1. These genes showed up regulation fold changes ranging from 1.1 to 66.35. Notable down regulated genes included brevican and FGF 5 (with fold changes of 1.3 and 1.4, respectively).

### Neurotrophin-, nerve-, and pain-related gene, and disc gene expression patterns in the annulus: expression patterns in herniated compared to Non-herniated discs

Although not related to discogenic low back pain, we were also interested in evaluating our data in terms of expression patterns which were significantly different in herniated discs vs. non-herniated discs (Table 5).

Of high interest in Table 5 are a number of genes with high relevance to pain, neurotrophins and nerves. These include the following: Calcitonin gene related peptide (down regulated 1.2 fold). Upregulated genes included neuropilin 2, nerve growth factor, reticulon 4, roundabout axon guidance receptor; these genes were upregulated 1.5 to 5.58 fold.

A large number of genes in the ion transport grouping also showed significant changes in this analysis; 98 genes showed significant up regulation, and 39 significant down regulation (most data not shown).

Genes with specific high relationships to disc cell biology included these significantly upregulated genes: a number of the collagens, fibronectin 1, hypoxia inducible factors-1 and −2 (alpha subunit), latent TGF-ß binding protein 1, TGF-ß receptor 1, several of the mitogen-activated protein kinases, proteoglycan 4, SOD, and the apoptosis-associated genes caspase 6 and TNFRSF1A-associated via death domain.

### Immunohistochemical studies

**Figure 1:**
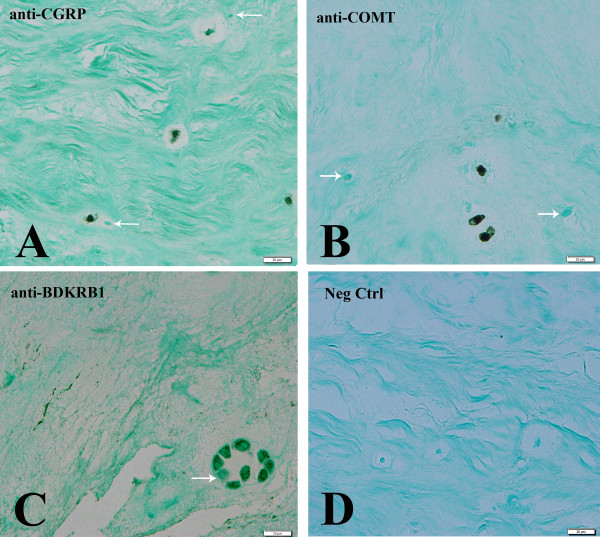
**Immunohistochemical localizations of calcitonin gene-related peptide, catechol-0-methyltransferase and bradykinin receptor B1:** Representative images showing localization of calcitonin gene-related peptide (Figure 1A), catechol-0-methyltransferase (Figure 1B) and bradykinin receptor B1 (Figure 1C) in annulus regions of the human disc. Figure 1D illustrates a negative control. Arrows mark nearby cells which did not show localization.

Paraffin-embedded annulus tissue was available for several of the subjects studied here (subjects # 5, 10, 14, 15, 19, 21 and 22 (Table 1) which enabled us to perform immunolocalization studies for products of three of the genes of special interest here (calcitonin gene-related peptide, catechol-0-methyltransferase and bradykinin receptor B1). For each of these immunolocalizations, cells were present with localization in single cells, clusters of cells, and both rounded and spindle-shaped cells in the outermost region of the annulus. Representative images are shown in Figures 1A-C; Figure 1D presents a negative control with the absence of any localization. Note that in Figures 1A-C adjacent cells were occasionally present which showed no localization.

## Discussion

In the present study we performed a genome-wide microarray analysis of human annulus tissue from patients with discogenic back pain compared to disc tissue from control subjects or compared to and herniated disc patients we had a special focus upon analysis of the expression of pain, nerve and neurotrophin-related genes. Ontology searches were an efficient search technique for identification of pain, and nerve-related genes [14]. Although there is a large body of clinical literature on low back pain, molecular studies are few, and those in the literature primarily focus upon population-based genetic studies of polymorphisms (SNPs) (see [13] for a review of pain and spinal disease). To the best of our knowledge, the present work is the first (non-SNP) genome-wide study of pain, neurotrophin and nerve-related genes in disc degeneration.

### Pain-related genes

Several well-recognized pain-related genes were found to have significant elevations in our analyses. Bradykinin receptor B1, calcitonin gene-related peptide and catechol-0-methyltransferase were significantly elevated in more degenerated discs (grade IV and V) compared to less degenerated (grades I-III) discs (Table 1). Bradykinin receptor B1 is formed after tissue injury and mediates hyperalgesia in chronic inflammation, but has very low expression in healthy tissue expression [16]. Calcitonin gene-related peptide (CGRP) has been found to be elevated in sensory nerves innervating inflamed tissue [17], in dorsal root ganglia and spinal cord in sciatic nerve injuries in the rat model by Orita et al. [18]. In the latter work, application or antibodies to nerve growth factor or its receptors TrkA or p75^NTR^ blocked CGRP expression. Catechol-*0*-methyltransferase (*COMT*) codes for a protein which is important in catabolic pathways of a number of pain-relevant neurotransmitters, including noradrenalin, adrenaline and dopamine [19]. In the present analyses comparing surgically operated discs to control discs (Table 4) and herniated to control discs, calcitonin gene-related peptide was the only one of these genes which was significant, and in these comparisons it was downregulated 1.2-fold. The COMT gene is very interesting since patients with a specific polymorphism identified by Zubieta et al. showed higher sensory and affective pain ratings [20].

Our ability to perform immunolocalization studies on human annulus tissues to determine the presence of bradykinin receptor B1, calcitonin gene-related peptide and catechol-0-methyltransferase (Figure 1) added strength to the present study, and confirmed the presence of products of these gene at the protein level within human disc tissue.

### Neurotrophin-related genes

Neurotrophins were also identified in the present analyses, including nerve growth factor in our studies of surgical vs. control discs, and herniated vs. control discs (significantly upregulated, Tables 4 and 5, respectively), and ciliary neurotrophic factor (down regulated, Table 4, surgical vs. control discs).

Studies have recently shown production of several neurotrophins by disc cells. Gigante et al. reported the presence of nerve growth factor (NGF) mRNA and the high affinity tyrosine kinase A receptor (trkA) and the low affinity p75 receptor in the rounded cells in the disc annulus and nucleus pulposus [21]. Recently Abe et al. reported on the expression of nerve growth factor (NGF) by human disc cells in control disc tissue in vivo and in vitro in cells from control discs using immunocytochemistry [22]. Nerve growth factor was found to be high in the outer annulus and herniated disc tissue. That work also demonstrated that the proinflammatory cytokines IL-1 and TNF-alpha had stimulatory effects on NGF. These authors suggested that such actions may play a role in nerve sprouting into the disc and may be associated with discogenic pain. Recent in vitro work from our lab has also confirmed that exposure of disc cells to IL-1ß in three-dimensional culture (which more accurately mimics the in vivo condition) results in elevated production of nerve growth factor by human annulus cells [23].

Ciliary neurotrophic factor whose expression is reported here, has not previously been known to be expressed in the human disc. Work from previous studies has shown that it can act as both a neuroprotective agent [24] and a trophic factor for motor neurons [25].

### Nerve-related genes

Several nerve-related genes should be mentioned in our analyses, including neuron navigator-1 and −2, neuropilin 2, reticulon 4 (neurite growth inhibitor), roundabout axon guidance receptor, homolog 3, and neural precursor cell expressed (developmentally down regulated 4-like) (Tables 3, 4 and 5). These genes also have not previously been known to be expressed in the human disc.

Neuron navigator 1 is a microtubule-associated protein involved in neuronal migration [26], and neuron navigator 2 is required for all-trans retinoic acid-mediated neurite outgrowth and axonal elongation [27]. Neuropilin 2 was significantly upregulated in all of our disc comparisons (Tables 3,4 and 5). Neuropilin 2 is another gene which we have found to be significantly upregulated in cultured annulus cells exposed to IL-1ß [23]. Neuropilin-1 and neuropilin-2 are membrane proteins implicated in aspects of neurodevelopment. They are semaphorin III receptors as shown by the work of Kolodkin et al. [28], and are expressed in overlapping populations of neurons in the embryonic nervous system. Semaphorin III is a protein which, when secreted in vitro, results in the collapse of neuronal growth cones and chemorepulsion of neurites. It is also needed for correct sensory afferent innervation [29]. Expression of this gene by annulus cells may provide evidence that annulus cells express this gene to block neurite ingrowth into the disc.

Roundabout (ROBO1) is a gene which encodes a receptor which is a member of the neural cell adhesion molecule family. It functions as an axon guidance receptor [30]. We found upregulation of this gene in surgical vs. control specimens (Table 4) and in herniated vs. control specimens (Table 5).

Reticulon 4 (neurite growth inhibitor) was identified with upregulation in each of our analyses, with 4.6 fold (Table 3, more degenerated discs vs. less degenerated discs), 5.96 fold (Table 4, surgical vs. control discs) and 5.58 fold (Table 5, herniated vs. control discs) changes. This gene, also called NOGO, is a neurite outgrowth inhibitor [31].

### Genes related to proinflammatory cytokines and chemokines

In Tables 3,4 and 5, in the section headed “Genes with special disc relevance” we have listed many proinflammatory cytokines and chemokines identified in our analyses. These deserve special mention here because a large number of proinflammatory cytokines are well recognized as products of disc cells themselves in vivo (see [32-36] for an introduction to this field). Many chemokines are also produced by disc cells (our unpublished data). It is very important to note in the present study that many proinflammatory cytokines and chemokines are now known to induce or exacerbate inflammatory and neuropathic pain and hyperalgesia [37-39]. We suggest here that this is an exceptionally important aspect of low back pain that has here-to-fore been little recognized. The degenerating human disc is primarily an avascular tissue site into which disc cells have contributed high levels of proinflammatory cytokines which are not cleared from the tissue and remain there over time. *We suggest that as nerves grow into the human annulus, they encounter a proinflammatory cytokine-rich milieu which exacerbates pain production.*

Kim et al. using in vitro work has suggested that disc cells themselves are involved in inflammatory activities, and suggested that interactions between annulus cells and nerve cells enhances the production of growth factors responsible for neovascularization and nerve ingrowth into the disc [40]. Previous research by Aoki et al. showed that disc inflammation potentially promoted axonal regeneration of dorsal root ganglion neurons innervating the disc in a rat model [41]. Using gene correlations, recent work by Lee et al. suggests that IL-1ß is generated during degeneration of the disc, and this stimulates expression of agents such as nerve growth factor, which result in nerve in growth into the disc [42].

It is important to recognize that proinflammatory cytokine production within the degenerating disc can also be exacerbated by repeated disc injury, which may lead to persistent proinflammatory cytokine elevation [43]; thus repeated disc injury may also influence neuroinflammation and pain.

A final important comment from the joint literature on proinflammatory mediators concerns the fact that release of these agents in damaged tissue and in the spinal cord is known to sensitize the peripheral terminals of nociceptors [44]. It is possible that similar hyper excitability of pain transmitting neurons results from proinflammatory cytokines in the disc matrix during degeneration; proinflammatory cytokines likely to be at play here are IL-1ß and TNF-α.

### Genes which share high importance to the disc itself

It was interesting that many genes were identified in our analyses which have relevance to the nerve, neurotrophin and pain ontology and to disc biology itself (as shown in section C of Tables 3,4 and 5). These included extracellular matrix (ECM) components, such as collagens, fibronectin, laminin, thrombospondin, brevican, proteoglycans, and versican, and also genes related to matrix degradation (metallopepdiases and TIMP metallopeptidase inhibitors), growth factors (connective tissue growth factor), nitric oxide synthase 3 (ENOS), SOD, and hypoxia inducible factor 1 alpha subunit. Also important to disc biology were the vitamin D receptor gene, growth arrest specific genes (important to cell senescence), apoptosis-related genes, and BMP receptor type II. Although some of these gene products may be resident only in sites of neurovascular ingrowth, many may influence the disc ECM itself and disc cell functions. Such findings point to the importance of future research directed towards identifying functional interactions between disc and nerve cells in vivo and in vitro. For example, it has long been recognized that there is an accumulation of fibronectin fragments in the aging/degenerating disc, and these fragments initiate signaling pathways which can increase MMP expression causing a cycle of matrix destruction [45-50]. Strong up regulation of fibronectin was present in our analyses (16.8 fold upregulation in more degenerated vs. healthier discs (Table 3), 37.0 fold upregulation in surgical vs. control discs (Table 4), and a 52.4 fold up regulation in herniated vs. control discs (Table 5).

Possible limitations to the present analyses include the fact that some specimens, noted as “controls” in Table 1, were obtained from the Cooperative Human Tissue Network (CHTN). Although these were shipped quickly to our lab post-autopsy as quickly as possible via CHTN, delays might result in potential alterations in mRNA levels during our study. The reader should note that in order to carefully investigate this issue, in Table 4 we compared findings in surgical specimens vs. those obtained from CHTN. As would be expected in different sized microarray group comparisons with ontology searchers, Table 4 does differ in some respects from data presented in Table 3. It is also worth noting that in our laboratory, CHTN specimens are also routinely used for derivation of disc cells in vitro, and in our hands over 98% of CHTN specimens yielded viable cells.

Another point for the reader to note is that our tables have reported gene expression fold changes which in some cases were less than 2.0, a level which is commonly used. Since we feel that smaller changes in important genes may be clinically relevant, we have reported these changes in our data tables.

## Conclusions

Even though our three study groups were not large, the present analysis showed that microarray analysis could successfully be used to examine key pain-, neurotrophin- and nerve-related genes in specimens of human disc tissue. Many genes were found in these ontology searches which held significance not only for nerves, pain and neurotrophins, but also for disc ECM, signaling and functional components. Key findings included confirmation of the presence of calcitonin gene-related peptide, catechol-0-methyltransferase and bradykinin receptor B1 at the protein level in the human annulus using immunohistochemistry, and identification of significant changes in a number of proinflammatory and chemokine genes identified from nerve, neurotrophin and pain ontology searches. Since the disc is primarily avascular, and since disc cells themselves produce proinflammatory cytokines and chemokines which are not removed from the tissue, we hypothesize that as nerves grow into the human annulus, they encounter a proinflammatory cytokine-rich milieu which may sensitize nociceptors and exacerbate pain production. Findings reported here point to the importance of future studies of the functional interactions between disc and nerve cells in vivo and in vitro.

## Methods

### Clinical study population

Experimental study of human disc specimens was approved prospectively by the authors’ Human Subjects Institutional Review Board. The need for informed consent was waived by the ethical board since disc tissue was removed as part of routine surgical practice. Scoring of disc degeneration utilized a modification of the Thompson scoring system [51] incorporating author ENH’s radiologic, MRI and surgical findings. The Thompson system scores disc degeneration over the spectrum from a healthy disc (Thompson grade I) to discs with advanced degeneration (grade V, the most advanced stage of degeneration) [51]. Patient specimens were derived from surgical disc procedures performed on individuals with herniated discs and degenerative disc disease. Surgical specimens were transported to the laboratory in sterile tissue culture medium. Non-surgical, control donor disc specimens were obtained via the National Cancer Institute Cooperative Human Tissue Network (CHTN); they were shipped overnight to the laboratory in sterile tissue culture medium and processed as described below. Specimen procurement from the CHTN was included in our approved protocol by our human subjects Institutional Review Board.

### Microarray analysis

Disc tissue was snap frozen in liquid nitrogen, pulverized (BioPulverizer, BioSpec Products, Inc., Bartlesville, OK, USA), and homogenized via the FastPrep-24 instrument (MP Biomedicals L.L.C., Santa Ana, CA, USA). Total RNA was isolated via a modified version of TRIzol Reagent (Life Technologies: Invitrogen, Carlsbad, CA, USA), and prepared for microarray hybridization using the GeneChip 3’ IVT Express Kit (Affymetrix, Santa Clara, CA). In brief, total RNA was reverse transcribed to synthesize cDNA, converted to double stranded DNA, subjected to transcription generating biotin-labeled amplified RNA (cRNA) and hybridized to the DNA microarray in the Affymetrix Fluidics Station 400. Affymetrix human U133 X3P arrays were used. The GCOS Affymetrix GeneChip Operating System (version 1.2, Affymetrix, Santa Clara, CA 95051) was used for determining gene expression levels. mRNA from annulus tissue from each subject was analyzed separately (i.e., samples were not pooled).

### Statistical analysis

The GCOS Affymetrix GeneChip Operating System (version 1.2, Affymetrix, Santa Clara, CA) was used for determining gene expression levels. GeneSifter^TM^ web-based software (VizX Labs, Seattle, WA, USA) was used to analyze all microarray data. Using GC-RMA (Robust multi-array average), Affymetrix ‘.cel’ files were uploaded to the GeneSifter^TM^ web site and normalized, and corrected for false discovery rate (FDR). Using the student t-test (2 tailed, unpaired), statistical significance was determined (p < 0.05).

*Gene Ontology (GO) searches* were employed in our analyses to select genes of interest and groups of critically important genes. This approach lets one avoid searching through results gene by gene, and provides a controlled vocabulary of search terms for gene characteristics. In our analyses, Gene Ontologies (GO) were generated by GeneSifter^TM^ based on the Gene Ontology Consortium. Searches were performed in the present study on “pain” and “nerve”; for each, ontologies were searched under “biological process”, “molecular function” (the activities of the gene product at the molecular level), and “cellular component” (parts or cells or the extracellular milieu). To aid the reader in visualizing the key terms covered in these ontology grouping, details are provided in Table 2.

Gene array data for the human disc specimens analyzed here have been uploaded to the Gene Expression Omnibus (GEO) website [GEO:GSE23130] and may be accessed there.

### Immunohistochemistry

*Bradykinin receptor B1 (BDKRB1) and calcitonin gene related peptide (CGRP) Immunohistochemistry:* Disc specimens were fixed in 10% neutral buffered formalin, embedded undecalcified, paraffin sections cut at 4 μm, collected on PLUS slides(Cardinal Health, Dublin, OH) and dried at 60°C. Sections were deparaffinized in xylene (Cardinal) and rehydrated through graded alcohols (AAPER, Shelbyville, KY) to distilled water. Antigen retrieval was performed using Biocare Antigen Decloaker Solution, pH 6.0 (Biocare Medical, Concord, CA) for 20 minutes at 95°C followed by cooling for 20 minutes. The remainder of the procedure was performed using the Dako Autostainer Plus (Dako, Carpenteria, CA) automated stainer. Endogenous peroxidase was blocked using 3% H_2_0_2_ (Sigma, St Louis, MO). Slides were incubated for 30 minutes with Serum-Free Protein Block (Dako); blocking solution was drained from slides and primary antibody applied. Sections were incubated for one hour with anti-Bradykinin receptor B1 (BDKRB1) (Novus Biologicals, Littleton, CO) at a 1:50 dilution, or with for one hour with anti-calcitonin gene related peptide (CGRP) (Abcam, Cambridge, MA) at a 1:100 dilution. Secondary antibody was 4 + Biotinylated Universal Goat Link (Biocare) for 10 minutes followed by 4+ streptavidin HRP Label (Biocare) for 10 minutes and DAB (Dako) for 5 minutes. Slides were removed from stainer, rinsed in water, counterstained with light green, dehydrated, cleared and mounted with resinous mounting media. Universal Rabbit Negative (Dako, Carpinteria, CA) was used as a negative control.

*Catechol-O-methyltransferase (COMT) immunohistochemistry* did not require antigen retrieval. Sections were prepared as described above, and incubated for one hour with anti-catechol-O-methyltransferase (COMT) (Lifespan Biosciences, Seattle, WA) at a 1:200 dilution. The secondary antibody and negative control utilized were as described above.

Positive control human tissues were also included with each immunolocalization run; for bradykinin receptor B1 this was brain, for calcitonin gene related peptide this was thyroid, and for catechol-O-methyltransferase, adrenal.

## Abbreviations

GO: Gene ontologies; GC-RMA: Robust multi-array average; HIF1: Hypoxia inducible factor 1; TGF-ß: Transforming growth factor beta; TIMP: Tissue inhibitor of metalloproteinases; CHTN: Cooperative Human Tissue Network; TNFα: Tumor necrosis factor-alpha; SNP: Single nucleotide polymorphism; CGRP: Calcitonin gene-related peptide; COMT: Catechol-*0*-methyltransferase; NGF: Nerve growth factor; IL-1: Interleukin-1; SOD: Superoxide dismutase; ECM: Extracellular matrix.

## Competing interests

The authors declare that they have no competing interests.

## Authors’ contributions

HEG and ENH are responsible for study concept and design. ENH contributed surgical disc specimens and disc grades. GLH performed gene searches and analyses. JAI performed immunohistochemistry. HEG identified critical genes and wrote the manuscript, and all authors approved the final manuscript.
